# Whole-cell catalyze L-dopa to dopamine via co-expression of transport protein AroP in *Escherichia coli*

**DOI:** 10.1186/s12896-023-00794-6

**Published:** 2023-08-29

**Authors:** Siyuan Gao, Ding Ma, Yongtao Wang, Alei Zhang, Xin Wang, Kequan Chen

**Affiliations:** State Key Laboratory of Materials-Oriented Chemical Engineering, College of Biotechnology and Pharmaceutical Engineering, Nanjing Tech University, Nanjing, 211816 Jiangsu China

**Keywords:** Dopamine, Whole-cell catalysis, Bio-catalysis, Transport proteins

## Abstract

Dopamine is high-value compound of pharmaceutical interest, but its industrial scale production mostly focuses on chemical synthesis, possessing environment pollution. Bio-manufacturing has caused much attention for its environmental characteristic. Resting cells were employed to as biocatalysts with extraordinary advantages like offering stable surroundings, the inherent presence of expensive cofactors. In this study, whole-cell bioconversion was employed to convert dopa to dopamine. To increase the titer and yield of dopamine production through whole-cell catalysis, three kinds of aromatic amino acid transport protein, AroP, PheP and TyrP, were selected to be co-expressed. The effects of the concentration of L-dopa, pyridoxal-5’- phosphate (PLP), reaction temperature and pH were characterized for improvement of bioconversion. Under optimal conditions, dopamine titer reached 1.44 g/L with molar yield of 46.3%, which is 6.62 times than that of initial conditions. The catalysis productivity of recombinant *E. coli* co-expressed L-dopa decarboxylase(DDC) and AroP was further enhanced by repeated cell recycling, which maintained over 50% of its initial ability with eight consecutive catalyses. This study was the first to successfully bioconversion of dopamine by whole-cell catalysis. This research provided reference for whole-cell catalysis which is hindered by cell membrane.

## Introduction

Dopamine [2-(3,4-Dihydroxyphenyl-D3)ethylamine or 3,4-dihydroxytyramine] is a high-value compound in pharmaceutics and materials [1]. As a neurotransmitter, dopamine plays a key role in learning and motivation [2]. As therapeutic agents, dopamine was taken by Arvid Carlsson for Parkinson’s disease [3]. Dopamine and its derivative norepinephrine and epinephrine are key therapeutic uses, like in emergency of COVID-19 [4, 5]. As a monomer of polydopamine (PDA), PDA materials have been applied in various fields [6–8]. Given the significance of dopamine in pharmaceutical and chemical industries, methods of dopamine synthesis have been studied but only for chemical synthesis [1]. Bio-manufacturing of dopamine was seldom reported [9], so an efficient bioconversion of dopamine is desired. In previous study, a pyridoxal, 5’-phosphate-dependent enzyme, as known as L-dopa decarboxylase (DDC, EC 4.1.1.28), was found that can catalyze L-dopa to form dopamine [10, 11]. Therefore, a DDC from *Harmonia axyridis* was employed in our previous research [12].

Whole-cell bioconversion has been a promising method for producing fine chemicals or pharmaceutical products from bulk chemicals with high selectivity. In biotransformation, whole-cell catalysis can effectively save energy by avoiding cell disruption and enzyme purification, provide the generation of cofactors and a stable and mild environment [13–16].However, permeability of cell membrane restricts the productivity of whole-cell bioprocess including fermentation, bioremediations and bio-catalysis [17]. Scientists and researchers, therefore, are focusing on the problem and proposed a range of strategies [18], including solvent and detergent treatment [19–22], freeze and thaw method [23]. These solutions maybe simple and effective but they are empirical and unsuitable for large scale for the inevitably extra steps [17]. The novel strategies arose with development of synthetic biology, such as cell surface display [24, 25] and modifying out membrane structures [18].

In this study, physical, chemical and biological methods were attempted to enhance the bioconversion rate of L-dopa to dopamine. Different transport proteins were selected and studied to enhance the productivity of whole-cell catalysis of L-dopa to dopamine. Then, the recombinant *E. coli* with AroP and DDC was constructed for efficient whole-cell bioconversion of dopamine and the reaction pH, temperature, the concentration of PLP and L-dopa were optimized. Finally, to enhance utilization efficiency of resting cells, the time of recover cells was also examined.

## Materials and methods

### Strains and media

The plasmids and strains used in this study were listed in Table 1. The strains were all cultured in Luria-Bertani (LB) medium consisting of 10 g/L peptone, 5 g/L yeast extract and 5 g/L sodium chloride with antibiotics. LB solid medium was extra added 4 g/L agar in LB medium. The concentration of ampicillin (Amp) and kanamycin (Kan) were 100 mg/L and 50 mg/L, respectively.

**Table 1:** Plasmids and strains used in this study

Strains and plasmids	Description	Source
Plasmids		
pET28a	Expression vector, Km^R^, P_T7_	Novagen
pRSFDuet -*pelB*	Expression vector, Amp^R^, P_T7_	This study
pRSFDuet	Expression vector, Amp^R^, P_T7_	This study
pET28a-DDC	Gene DDC inserted between *EcoRI* and *HindIII* sites of pET28a	This study
pRSFDuet-pelB-*aroP*	Gene *aroP* inserted by infusion clone in pRSFDuet -pelB	This study
pRSFDuet-*aroP*	Gene *aroP* inserted by infusion clone in pRSFDuet	This study
pRSFDuet-*pheP*	Gene *pheP* inserted by infusion clone in pRSFDuet	This study
pRSFDuet-*tyrP*	Gene *tyrP* inserted by infusion clone in pRSFDuet	This study
*E. coli* strains		
BL21(DE3)		Invitrogen
BL21-28a-DDC	*E. coli* BL21(DE3) harboring plasmid pET28a-DDC	This study
BL21-AB-*aroP*	*E. coli* BL21(DE3) harboring plasmid pRSFDuet-*pelB*-*aroP* and pET28a-DDC	This study
BL21-AD-*aroP*	*E. coli* BL21(DE3) harboring plasmid pET28a-DDC and pRSFDuet-*aroP*	This study
BL21-AD-*pheP*	*E. coli* BL21(DE3) harboring plasmid pET28a-DDC and pRSFDuet-*pheP*	This study
BL21-AD-*tyrP*	*E. coli* BL21(DE3) harboring plasmid pET28a-DDC and pRSFDuet-*tyrP*	This study

### Construction of plasmids and expression of proteins

The gene *arop* (gene ID: 946,018), *phep* (gene ID: 945,199), *tyrp* (gene ID: 946,412) are all belong to aromatic amino acid symporter of *E. coli* and were amplified by polymerase chain reaction. The constructed genes were inserted in plasmid vectors by homologous recombination. The primers with homologous fragments are listed in Table 2 and sequences of homologous arms were underlined. All the recombinant strains were inoculated from a freshly transformed single colony to 5 mL of LB medium and seeded into 100 mL of LB medium after cultivation for 12 h at 37 ℃. 50 mg/L kana was added into medium used for culturing BL21-28a-DDC. 50 mg/L ampicillin and 25 mg/L kanamycin were added into medium used for culturing BL21-AB-*aroP*, BL21-AD-*aroP*, BL21-AD-*pheP* and BL21-AD-*tyrP*. Cell density was monitored by OD_600_. When OD_600_ reached 0.6–0.8, 0.5 mM IPTG was added into medium for induction. After induction, the cells were cultured at 25 ℃ for 10 h. Cells were harvested by centrifugation (6000 rpm, 10 min, 4 ℃) and resuspended in various PBS buffers of pH (5.7-8). The primers were listed in Table 2.

**Table 2:** Primers used in this study

Recombinant plasmids	Forward primer	Reverse primer
pRSFDuet-*Arop*	TTAATAAGGAGATATACCATGATGGAAGGTCAACAGCACGGC	TTATGCGGCCGCAAGCTTTTAATGCGCTTTTACGGCTTTGGC
PRSFDuet-*pelB*-*Arop*	TTAATAAGGAGATATACCATGAAATACCTGCTGCCGAC	TTATGCGGCCGCAAGCTTTTAATGCGCTTTTACGGCTTTGGC
pRSFDuet-*Phep*	TTAATAAGGAGATATACCaTGAAAAACGCGTCAACCGTATCG	TTATGCGGCCGCAAGCTTTTATTTCCGACGCAGCGTTTTAAA
pRSFDuet-*Tyrp*	TTAATAAGGAGATATACCaTGAAAAACAGAACCCTGGGAAGT	TTATGCGGCCGCAAGCTTTCACCCCACTTCTGGTAACAACCC

Sequence of homology arms were underlined

### Whole-cell bioconversion of dopamine from L-dopa

#### Production of cells for whole-cell catalysis

All recombinant *E. coli* cells mentioned above were inoculated into 5 mL LB medium from a single colony in LB solid medium and cultured at 37 ℃ and 200 rpm. 0.5 mM IPTG was added into the medium when OD_600_ reached 0.6–0.8. After cultured at 25 ℃ for 12 h, the cells were harvested by centrifugation of 6000 rpm at 4 ℃, washed and re-suspended in PBS buffer.

#### Pre-requisite experiment of whole-cell transformation

To determine whether the cell membrane is a key factor which inhibited the efficiency of catalysis, the pretest was carried out in a 10 mL tube. Tween-20, tween-60, tween-80, cetyl trimethyl ammonium bromide (CTAB), dimethyl sulfoxide (DMSO) and ionic liquid (molar ration of choline chloride and glycerol was 1:1) were added into reaction system to investigate the effects of surfactants on catalysis. The reaction system consisted of 0.5 g/L L-dopa, 0.4 mM PLP, 1‰ (v/v) surfactant mentioned before, resting cells (OD_600_ = 10) and 100 mmol/L PBS buffer (pH = 7.0).

#### Study of the optimum catalysis conditions

To find out the optimum catalysis conditions, the whole-cell catalysis was performed in a 10 mL tube and reaction system consisted of L-dopa, PLP, resting cells (OD_600_ = 10) and 100 mmol/L PBS buffer with univariate research. The key factors in this study included kinds of transport protein and plasmid vector, reaction temperature, pH, concentration of L-dopa and PLP. (1) Three transport proteins were Arop, PheP and TyrP and cloned in pRSFDuet. (2) The plasmids, pRSFDuet-pelB (pelB was cloned from pET22b) and pRSFDuet, were used to confirm the function of signal peptide pelB. (3) L-dopa of 1, 2, 3, 4 g/L was added into system with 0.4 mmol/L PLP under 37 °C at pH 7.0. (4) The concentration of PLP was between 0 and 1 mmol/L and the conditions of reaction were 4 g/L L-dopa under 37 °C at pH 7.0. (5) Reaction temperatures were set from 40 ℃ to 70 ℃ with interval of 5 °C. The system consisted of 4 g/L L-dopa without PLP at pH 7.0. (6) Reaction pH controlled by different ratio of solution Na_2_HPO_4_ and solution NaH_2_PO_4_ from 5.7 to 8.0 under 50 °C and L-dopa was 4 g/L without PLP. All reaction times were controlled within 1 h.

#### Repeating whole-cell catalysis with recover cells

As determined the condition of catalysis, the recycle times of whole-cell bioconversion was investigated at 40 ℃, 45 ℃ and 50 ℃. The recombinant *E. coli* BL21-AB-*aroP* was cultured and cells were obtained by the method mentioned in 2.3.1. The reaction system included 4 g/L L-dopa, cells (OD_600_ = 10) and PBS buffer (pH = 7.0) and reaction lasted for 1 h each time. The biotransformation continued to circulate until no dopamine was detected under one temperature conditions.

### Analysis methods

The cell growth was monitored by cell density (OD_600_). L-dopa and dopamine was detected by high-performance liquid chromatography (HPLC) system (Agilent 1260, USA) equipped with a TC-C18 column (150 mm × 4.6 mm, 5 μm, USA). Detecting temperature was 25 ℃ and detecting wavelength was 280 nm with a UV detector. 1 mL/min mobile phase was a solution of 0.1% TFA solution - acetonitrile (96:4). All experiments were carried out in at least triplicate.

## Results and discussion

### Expression of transport protein to enhance the biotransformation of L-dopa to dopamine

**Fig. 1:**
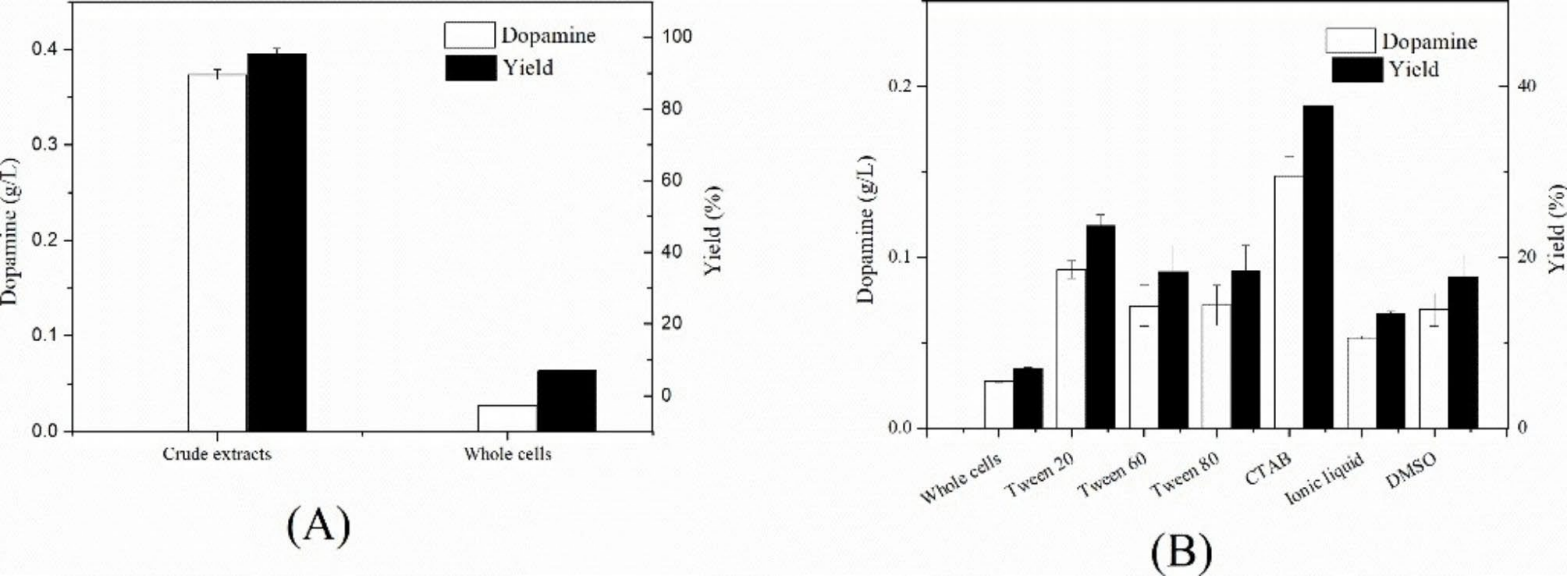
Different forms of catalysis to producing dopamine **(A)** Comparison of crude extracts and whole cell catalysis; **(B)** Effects of different surfactants on whole-cell catalysis

As is shown in Fig. 1(A), the titer and yield of dopamine catalyzed by crude extracts was 0.37 g/L and 95.47%, respectively and 13.66 times higher than that catalyzed by resting cells. However, the process for acquisition of crude extracts needed cell disruption. Meanwhile, PLP, an expensive cofactor, is necessary for crude extracts catalysis. Taking two points into consideration, the cost of crude extract catalysis is much higher than whole-cell catalysis. Thus, it is worth developing a strategy for the synthesis of dopamine by whole cell catalysis. To illustrate the disparity between crude extract catalysis and whole-cell catalysis, we speculated that the cell membrane hindered the transfer of products and substrates. To validate the suspect, six kinds of surfactant were selected and added into reaction. All surfactants contributed to bioconversion of L-dopa to dopamine with minimum of 1.9 times that the yield of dopamine catalyzed by adding surfactants increased by. Among six kinds of surfactant tested, the outcome of CTAB was the most outstanding and the titer of dopamine was increased by a factor of 5.4 compared with that merely catalyzed by resting cells. This phenomenon was also found by Zhao that *E. coli* cultures were treated with 2% (v/v) solutions of eight surfactants for 30 min and the effect of CTAB was excellent[22]. A similar case was that citric acid production was increased by 1.4–1.8 times for *Yarrowia lipolytica* strains by addition of Triton X-100, a surfactant [26]. Therefore, the dominating reason was the membrane permeability to L-dopa that caused a descend in catalysis efficiency by whole-cell catalysis compared to crude extract catalysis.

To solve the membrane permeability, engineering modification on cells was conducted, such as expressing transport protein. In fact, five systems of aromatic amino acids transport had been reported in *E. coli* [27]. Due to structural similarity to L-dopa, we selected phenylalanine, tyrosine transport protein for further research. Additionally, a general aromatic transport protein was also employed into research. General transport system was coded by *aroP* [28] and the tyrosine- and phenylalanine-specific systems were coded by *tyrP* and *pheP* in this study [29].The results in Fig. 1(B) obviously illustrated that cell membrane did inhibit transfer of L-dopa and dopamine, resulting in poor performance of bioconversion. To decrease the substrate diffusion barrier, an efficient transport system was constructed that three kinds of transport protein, AroP, TyrP and PheP contributed to transfer substrate into cell. AroP, TyrP and PheP were aromatic amino acid symporter in *E. coli*. AroP and PheP belong to the amino acid-polyamine-organocation (APC) superfamily (https://www.uniprot.org/uniprot/P15993). PheP belongs to the amino acid/polyamine transporter 2 family (https://www.uniprot.org/uniprot/P0AAD4) [27, 28].

**Fig. 2:**
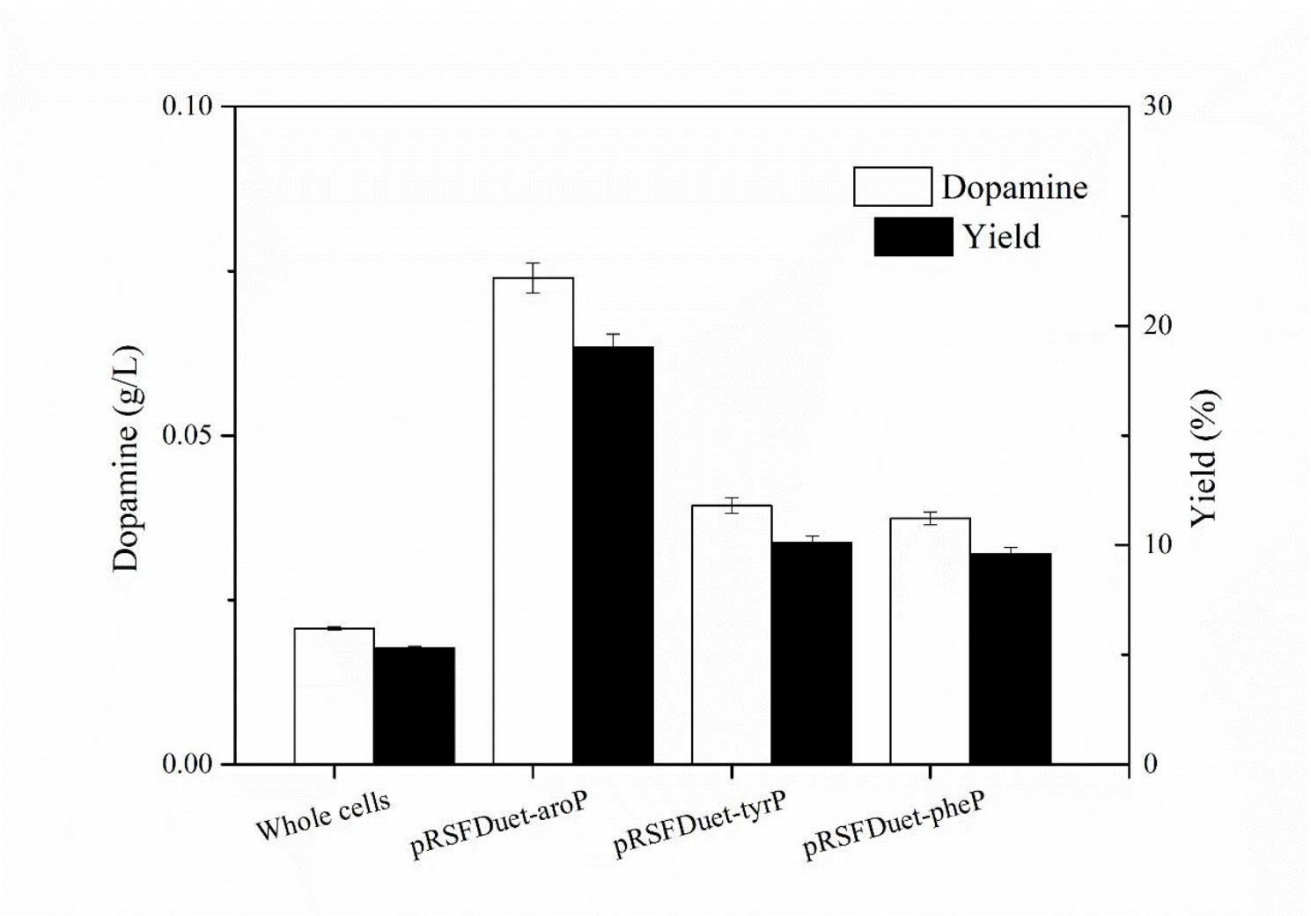
Effects of different transporters on catalysis

In order to investigate the influences of transport proteins on catalysis efficiency, the coding genes of three transport proteins were cloned into pRSFDuet and co-transformed with pET28a-DDC into BL21(DE3) to form recombinant cell BL21-AD-*aroP*, BL21-AD-tyrP and BL21-AD-pheP. In Fig. 2, all three transport protein had enhanced the production of dopamine and the most obvious result was BL21-AB-*arop* that titer and yield of which had up to 74 mg/L and 19%, respectively was nearly 4 times than that of whole-cell catalysis. According to Shang et al., AroP exhibited high affinity to phenylalanine and tyrosine similar with L-dopa [30]. Taking transport protein to enhance production is an effective strategy. The yield of L-tryptophan has been improved by 12.6% through modification of tryptophan transport system by Liu et al. [31]. The highest cadaverine production at that time was obtained by Ma et al. using recombinant *E. coli* co-overexpressing CadA and CadB which was a lysine/cadaverine transport antiporter [32].

**Fig. 3:**
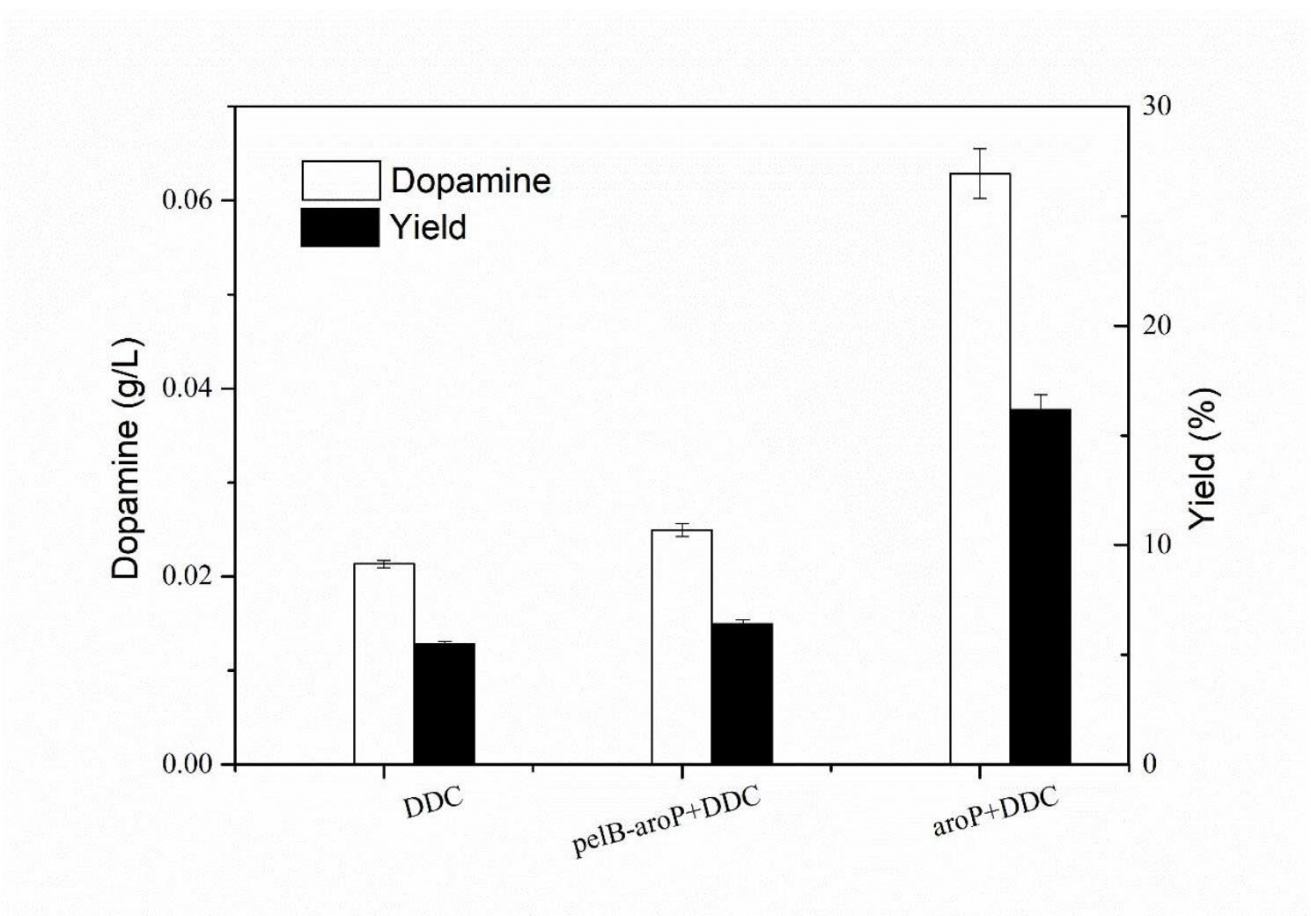
Effects of *pelB* on catalysis

Considering that transport protein was used to help transportation, pRSFDuet-pelB harboring a signal peptide pelB from pET22b, which can enhance transcript and translation levels of genes and direct protein translocation, was used as a vector to expression of AroP protein [33, 34]. BL21(DE3) harboring pRSFDuet-pelB-*aroP* and pET28a-DDC was constructed as BL21-AB-*aroP*. As signal peptide, PelB was expected to help the AroP periplasm secretion. However, PelB did not achieve the desired effect and the yield of dopamine was half of BL21-AD-*aroP* (Fig. 3). The result was contrary to that reported by Ma et al., where CadB fused to pelB increased the cadaverine production [32].Unlike CadB structure that expression and translocation of CadB to cell envelope contributed to L-lysine/cadaverine exchange and enhance production of cadaverine [35], *aroP* is highly hydrophobic with transmembrane potential so AroP fused with pelB may make activity decreased [30]. Thus, BL21-AD-*aroP* was determined to further study to enhance bioconversion of dopamine from L-dopa.

### Optimizing BL21-AD-***aroP*** catalysis conditions

**Fig. 4:**
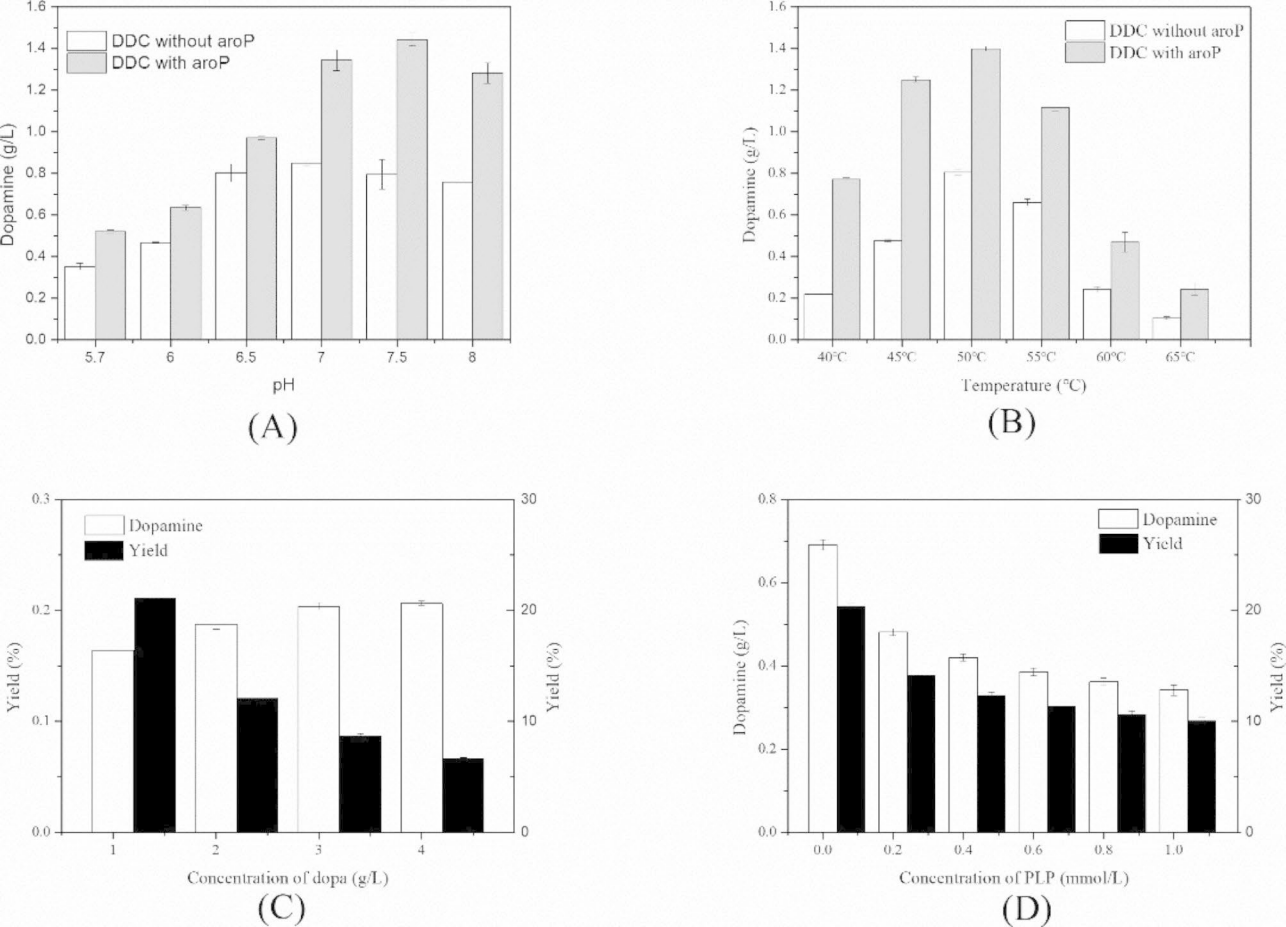
Optimization of whole-cell catalysis **(A)** Effects of pH on reaction **(B)** Effects of temperature on reaction **(C)** Effects of dopa concentration on reaction **(D)** Effects of PLP concentration on reaction

BL21-AD-*aroP* harboring pET28a-DDC and pRSFDuet-*aroP* conding dopa decarboxylase DDC and transport protein AroP, respectively. In order to increase the titer and yield of dopamine catalyzed by BL21-AD-*aroP*, the catalysis conditions including reaction temperature and pH and concentration of L-dopa and PLP were examined. Six pH gradients, 5.7, 6.0, 6.5, 7.0, 7.5 and 8.0, were set to study the effect of pH on catalysis. The bioconversion lasted for 1 h at 45 ℃ with 4 g/L substrate. Similar to results of temperature, the titer of dopamine was more in the presence of AroP (Fig. 4A). The highest titer was found at pH 7.5 instead of 7.0. The phenomenon would be related to nature of AroP because in absence of AroP, the peak value was at pH 7.0. In addition, the most suitable pH for DDC was 7.5 in our previous study [12]. After optimizing conditions of reaction, the highest yield was up to 46.2% and the productivity of catalysis under optimal conditions had been raised 8.66 times against the initial condition.To investigated effects of temperature on whole-cell catalysis, the reaction temperature ranges from 40 ℃ to 70 ℃, every 5 degrees as a group with system consisting of 4 g/L dopa and pH of 7.0. PBS buffer and catalysis lasted for 1 h. In general, whole-cell catalysis with AroP was superior to that without AroP and effect at 45 ℃ was the most salient. The titer of dopamine by expressing AroP was 3.5 times of that of without expressing AroP. The highest yield was up to 45.1% (Fig. 4B). In addition, the concentration of substrate, L-dopa, was also investigated. The concentration of L-dopa was set as 1, 2, 3 and 4 g/L, respectively. The reactions were performed under conditions of pH = 7.0, 40 ℃ and 1 mM PLP. The results were shown in Fig. 4C that titer slightly increased but yield gradually decreased with improvement of dopa concentration. The same phenomenon was also found in purified catalysis [12]. The phenomenon further reflected that although AroP had been overexpressed, the reaction rate was quite low in whole-cell transformation which means a huge room for improvement of bioconversion of dopamine.

PLP as a key cofactor for dopa decarboxylase, was essential in the reaction. Extra 0, 0.2, 0.4, 0.6, 0.8 and 1 mM PLP was added into system consisting of 1 g/L dopa and pH = 7.0 PBS buffer and catalysis was at 40 ℃ for 1 h. According to Fig. 4D, additional PLP did not enhance the titer and yield of biotransformation and inhibited the catalysis instead. PLP synthesized by *E. coli* itself was enough for catalysis and extra addition PLP could be adverse. To account for this result, the initial cellular PLP was enough for poor activity of whole-cell catalysis and extra PLP made the environment more adverse leading to decrease the productivity [36].

### Cycle catalyzing by BL21-AD-***aroP***

**Fig. 5:**
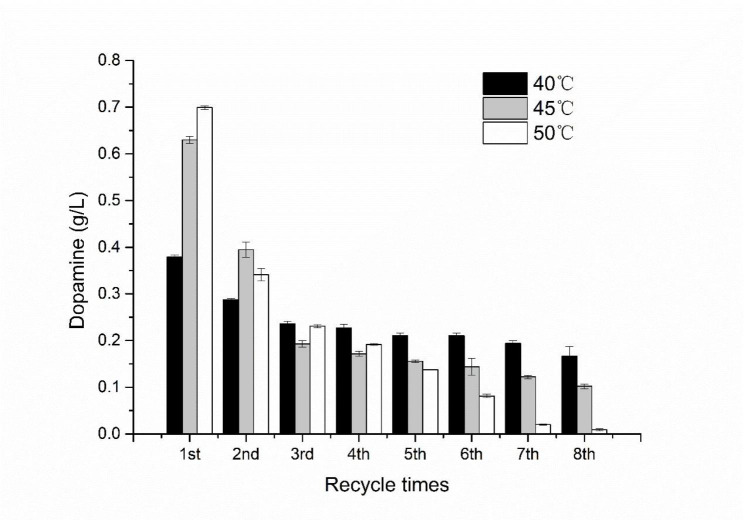
Dopamine productivity of the repeated cell recycling processes under 40 °C, 45 °C and 50 °C

Although the yield of crude extract catalysis was high, there was a problem of rapid loss of enzyme activity leading to discontinuous catalysis. On the contrary, although whole-cell catalysis was slow and yield was not high, it can be reused to improve the utilization rate of the enzyme. Thus, cells in catalysis system was recycled by centrifugation after bioconversion and was used for the next round of catalysis. Total 8 rounds of catalysis with 2 g/L substrate was taken to investigate the performance of cell cycle catalysis and the reactions took place at 40, 45 and 50 ℃, respectively. The results were shown in Fig. 5 that catalytic property under three temperature conditions was different. Under condition of 50 ℃, the titer of first catalysis was the highest but titer nearly disappeared at 8th catalysis, and catalytic performance was the fastest decrease among the three groups. The performance at 45 ℃ was similar to 50 ℃ but dopamine was still up to 0.1 g/L in the 8th catalysis, which was one quarter of the 1st catalysis. Compared to 45 and 50 ℃, the titer of first catalysis was the lowest but the 8th titer was the highest. Thus, cells at 40 ℃ possessed the most stable catalytic performance. The total titers and yield of dopamine were 1.91, 1.91 and 1.71 g/L and 11.9%, 11.9% and 10.7%, respectively in 8 rounds catalysis under 40, 45 and 50 ℃. Here, we attempted to co-express transport protein AroP and DDC for whole-cell catalyzing dopa to dopamine. Under optimal condition, 4 g/L of dopa was converted into 1.85 g/L of dopamine with a yield of 46.2%. After 8 rounds of cyclic catalysis, the catalytic performance of the resting cells remained above 40% catalytic performance of initial under the optimal condition. Given reaction lasted for only 1 h, the accumulation of dopamine did not reach the peak value. The titer and yield would further increase as time goes on.

To increase utilization of enzyme in bioconversion, immobilization was the first choice and had an excellent outcome. Zhou et al. reported that the immobilized ChBD-CadA can catalyze 200 g/L L-lysine to cadaverine of 135.6 g/L within 120 min and possess more than 57% activity after four cycles of use [37]. Compared to immobilized enzymes catalysis, whole-cell catalysis circumvented the need of process and materials of immobilization. At the same time, cell membrane shield enzyme from adverse surroundings and enable the resting cell to cycle catalysis [13]. But to overcome the disadvantages that poor utilization of cells limiting the further application of whole-cell bioconversion, repeated cell recycling is a conventional solution to get the utmost out of whole-cell catalysis [16]. Ying et al. reported that the titer of L-pipecolic acid reached 17.25 g/L under repeated cell recovery, which was 2.7 times of that without repeated cell recovery [16]. Cell recovering was not applied in whole-cell catalysis but also in fermentation. According to Ma, succinic acid productivity and mass yield was up to 1.81 g/L h and 0.85 g/g, respectively after three times of recycling cell [38]. In our study, we succeeded in cycle bioconversion by whole-cell catalysis that the catalysis activity was remained over 50% at 40 °C after eight batches of catalyses.

## Conclusion

In this study, we co-expressed dopa decarboxylase (DDC) and transport protein AroP in *E. coli* BL21(DE3) to enhance the titer and yield of dopamine production through whole-cell catalysis. The presence of permeability limited the efficiency of whole-cell catalysis. To solve the problem of permeability, AroP, PheP and TyrP were selected and expressed in *E. coli* BL21(DE3). AroP was the optimal transport protein whose coding gene was cloned into expression vector pRSFDuet. Additionally, reaction conditions were investigated to further enhance the efficiency of whole-cell catalysis. The best condition was conducted under 50 °C at pH 7.5 with 4 g/L of L-dopa. Compared with the initial catalytic results, the optimized productivity increased by 8.66 times. With the aim of maximization of cells utilization, repeated cell recovery was studied that the catalysis activity preserved over 50% at 40 °C after eight batches of catalyses. To the best of our knowledge, we are the first that successfully synthesized dopamine from L-dopa by whole-cell catalysis. This work also provides reference for whole-cell catalysis which is hindered by permeability.

## Data Availability

All data generated or analyzed during this study will be available from the first author (Siyuan Gao, 201,962,118,011@njtech.edu.cn) for anyone who wishes to access the data.

## Abbreviations

PLP: Pyridoxal-5’- phosphate
DDC: L-dopa decarboxylase
LB: Luria-Bertani
Amp: Ampicillin
Kan: Kanamycin
PDA: Polydopamine
CTAB: Cetyl trimethyl ammonium bromide
DMSO: Dimethyl sulfoxide
HPLC: High-performance liquid chromatography
APC: Acid-polyamine-organocation

## References

[1] Kurt AG, Aytan E, Ozer U, Ates B, Geckil H (2009). Production of L-DOPA and dopamine in recombinant bacteria bearing the *Vitreoscilla* hemoglobin gene. Biotechnol J.

[2] Berke JD (2018). What does dopamine mean?. Nat Neurosci.

[3] Carlsson A (2001). A half-century of neurotransmitter research: impact on neurology and psychiatry. Biosci Rep.

[4] Nataf S (2020). An alteration of the dopamine synthetic pathway is possibly involved in the pathophysiology of COVID-19. J Med Virol.

[5] Khalefah MM, Khalifah AM. Determining the relationship between SARS-CoV-2 infection, dopamine, and COVID-19 complications. J Taibah Univ Med Sci. 2020; November. 10.1016/j.jtumed.2020.10.006 PMC7646371 33173452

[6] Liu Z, Qu S, Weng J (1999). Application of polydopamine in surface modification of biomaterials. Biotechnol Bioeng.

[7] Geng W, Wang L, Jiang N, Cao J, Xiao Y-X, Wei H (2018). Single cells in nanoshells for the functionalization of living cells. Nanoscale.

[8] Wang G, Wang L, Liu P, Yan Y, Xu X, Tang R (2010). Extracellular silica Nanocoat confers Thermotolerance on Individual cells: a case study of material-based functionalization of living cells. ChemBioChem.

[9] Anderson WA, Moo-Young M, Legge RL (1992). Development of a multienzyme reactor for dopamine synthesis: I. Enzymology and kinetics. Biotechnol Bioeng.

[10] Gao S, Guo Y, Ma C, Ma D, Chen K, Ouyang P et al. Characterization and application of a recombinant dopa decarboxylase from *Harmonia axyridis* for the efficient biosynthesis of dopamine. Chin J Chem Eng. 2021.

[11] Ren LQ, Wienecke J, Hultborn H, Zhang M (2016). Production of dopamine by aromatic l-Amino acid decarboxylase cells after spinal cord Injury. J Neurotrauma.

[12] Wachtmeister J, Rother D (2016). Recent advances in whole cell biocatalysis techniques bridging from investigative to industrial scale. Curr Opin Biotechnol.

[13] Wang X, Cai P, Chen K, Ouyang P (2016). Efficient production of 5-aminovalerate from L-lysine by engineered *Escherichia coli* whole-cell biocatalysts. J Mol Catal B Enzym.

[14] Ying H, Chen X, Cao H, Xiong J, Hong Y, Bai J (2009). Enhanced uridine diphosphate N-acetylglucosamine production using whole-cell catalysis. Appl Microbiol Biotechnol.

[15] Ying H, Wang J, Wang Z, Feng J, Chen K, Li Y (2015). Enhanced conversion of L-lysine to L-pipecolic acid using a recombinant *Escherichia coli* containing lysine cyclodeaminase as whole-cell biocatalyst. J Mol Catal B Enzym.

[16] Chen RR (2007). Permeability issues in whole-cell bioprocesses and cellular membrane engineering. Appl Microbiol Biotechnol.

[17] Ni Y, Chen RR (2004). Accelerating whole-cell biocatalysis by reducing outer membrane permeability barrier. Biotechnol Bioeng.

[18] Liu Y, Hama H, Fujita Y, Kondo A, Inoue Y, Kimura A (1999). Production of S-lactoylglutathione by high activity whole cell biocatalysts prepared by permeabilization of recombinant *Saccharomyces cerevisiae* with alcohols. Biotechnol Bioeng.

[19] Fontanille P, Larroche C (2003). Optimization of isonovalal production from alpha-pinene oxide using permeabilized cells of *Pseudomonas rhodesiae* CIP 107491. Appl Microbiol Biotechnol.

[20] Silveira M, Jonas R (2002). The biotechnological production of sorbitol. Appl Microbiol Biotechnol.

[21] Zhao W, Huang J, Peng C, Hu S, Ke P, Mei L (2014). Permeabilizing *Escherichia coli* for whole cell biocatalyst with enhanced biotransformation ability from L-glutamate to GABA. J Mol Catal B Enzym.

[22] Matsumoto T, Takahashi S, Kaieda M, Ueda M, Tanaka A, Fukuda H (2001). Yeast whole-cell biocatalyst constructed by intracellular overproduction of *Rhizopus oryzae* lipase is applicable to biodiesel fuel production. Appl Microbiol Biotechnol.

[23] Sroga GE, Dordick JS (2002). A strategy for in vivo screening of subtilisin E reaction specificity in *E. coli* periplasm. Biotechnol Bioeng.

[24] Shimazu M, Mulchandani A, Chen W (2001). Cell surface display of organophosphorus hydrolase using ice nucleation protein. Biotechnol Prog.

[25] Mirbagheri M, Nahvi I, Emtiazi G, Darvishi F (2011). Enhanced production of citric acid in *Yarrowia lipolytica* by Triton X-100. Appl Biochem Biotechnol.

[26] Sarsero JP, Wookey PJ, Gollnick P, Yanofsky C, Pittard AJ (1991). A new family of integral membrane proteins involved in transport of aromatic amino acids in *Escherichia coli*. J Bacteriol.

[27] Brown KD (1970). Formation of aromatic amino acid pools in *Escherichia coli* K-12. J Bacteriol.

[28] West TP (1998). Isolation and characterization of an *Escherichia coli* B mutant strain defective in uracil catabolism. Can J Microbiol.

[29] Honoré N, Cole ST, Erratum (1990). Nucleotide sequence of the *arop* gene encoding the general aromatic amino acid transport protein of *Escherichia coli* k-12: homology with yeast transport proteins. Nucleic Acids Res.

[30] Liu Q, Cheng Y, Xie X, Xu Q, Chen N (2012). Modification of tryptophan transport system and its impact on production of L-tryptophan in Escherichia coli. Bioresour Technol.

[31] Ma W, Cao W, Zhang H, Chen K, Li Y, Ouyang P (2015). Enhanced cadaverine production from L-lysine using recombinant *Escherichia coli* co-overexpressing CadA and CadB. Biotechnol Lett.

[32] Lei SP, Lin HC, Wang SS, Higaki P, Wilcox G (1992). Characterization of the *Erwinia carotovora peh* gene and its product polygalacturonase. Gene.

[33] Sletta H, Tøndervik A, Hakvåg S, Vee Aune TE, Nedal A, Aune R (2007). The presence of N-terminal secretion signal sequences leads to strong stimulation of the total expression levels of three tested medically important proteins during high-cell-density cultivations of *Escherichia coli*. Appl Environ Microbiol.

[34] Soksawatmaekhin W, Uemura T, Fukiwake N, Kashiwagi K, Igarashi K (2006). Identification of the cadaverine recognition site on the cadaverine-lysine antiporter CadB. J Biol Chem.

[35] Ma W, Cao W, Zhang B, Chen K, Liu Q, Li Y (2015). Engineering a pyridoxal 5’ -phosphate supply for cadaverine production by using *Escherichia coli* whole-cell biocatalysis. Sci Rep.

[36] Zhou N, Zhang A, Wei G, Yang S, Xu S, Chen K (2020). Cadaverine Production from L-Lysine with chitin-binding protein-mediated lysine decarboxylase immobilization. Front Bioeng Biotechnol.

[37] Ma JF, Jiang M, Chen KQ, Xu B, Liu SW, Wei P (2010). Succinic acid production with metabolically engineered *E. coli* recovered from two-stage fermentation. Biotechnol Lett.

